# A case of a pulmonary‐renal syndrome caused by streptococcal infection

**DOI:** 10.1002/rcr2.1083

**Published:** 2023-01-10

**Authors:** Junki Shimokawa, Hiroaki Nagano, Yoshihiko Raita

**Affiliations:** ^1^ Department of General Medicine Mine City Hospital Mine‐shi Japan; ^2^ Department of Respiratory Medicine Okinawa Chubu Hospital Uruma‐shi Japan; ^3^ Department of Nephrology Okinawa Chubu Hospital Uruma‐shi Japan

**Keywords:** diffuse alveolar haemorrhage, methylprednisolone, post‐streptococcal glomerulonephritis, pulmonary‐renal syndrome, streptococcal infection

## Abstract

Pulmonary‐renal syndrome (PRS) is defined as a combination of diffuse alveolar haemorrhage and glomerulonephritis. An 18‐year‐old woman visited our hospital with a 2‐day history of fever, dyspnoea, and leg edema. Laboratory investigations revealed an elevated inflammatory reaction, increased serum creatinine levels, and normocytic anaemia. Additionally, the anti‐streptolysin‐O titre was positive, and complement component‐3 levels were decreased. Urinalysis revealed proteinuria and hematuria. Bronchoalveolar lavage aliquots were progressively more hemorrhagic. These findings supported a diagnosis of PRS secondary to streptococcal infection. The patient was treated with high‐dose methylprednisolone and antibiotics. After 4 days of treatment, her respiratory symptoms and serum creatinine levels improved. Steroid tapering was performed over 15 days. The findings in this case indicate that streptococcal infection is a potential cause of PRS, and that short‐term steroid therapy is an effective treatment.

## Graphical abstract

We report a case of a pulmonary‐renal syndrome caused by streptococcal infection. This report highlights the facts that streptococcal infection is a relatively rare cause of pulmonary‐renal syndrome, and short‐term high‐dose steroid monotherapy may be sufficient for its treatment.

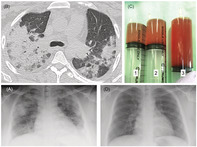

## INTRODUCTION

Pulmonary‐renal syndrome (PRS) is a clinical syndrome characterized by diffuse alveolar haemorrhage and rapidly progressing glomerulonephritis. Occasionally, PRS can lead to severe respiratory failure requiring intensive medical treatment. Autoimmune diseases, including Goodpasture syndrome, antineutrophil cytoplasmic autoantibody (ANCA)‐related vasculitis, and systemic lupus erythematosus, are common causes of PRS.^1^ However, streptococcal infections are rarely a cause of PRS.^2^, ^3^, ^4^ We report a case of PRS caused by streptococcal infection of the skin that was treated with antibiotics and short‐term steroid monotherapy.

## CASE REPORT

**FIGURE 1:**
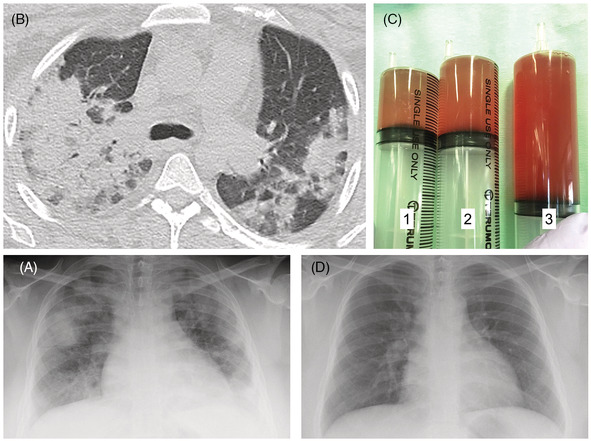
A chest radiograph showing an enlarged heart shadow and bilateral diffuse infiltrates with geographic distribution (A). Computed tomography shows multifocal diffuse consolidation and ground‐glass opacities predominantly in the upper lobes of the bilateral lungs (B). The red colour of the bronchoalveolar lavage fluid from the right upper lobe has intensified with each washing (C). After treatment with steroid therapy, a chest radiograph shows significant improvement (D). BAL, bronchoalveolar lavage

An 18‐year‐old female with uncontrolled atopic dermatitis and bronchial asthma presented to our hospital with a 2‐day history of fever, dyspnea on exertion, and leg edema. She denied hemoptysis or sore throat. She was using inhaled corticosteroids for bronchial asthma but no other medications or supplemental drugs. On arrival at the emergency room of our hospital, she was hypoxic (peripheral capillary oxygen saturation 86% at 4 L/min nasal oxygen), tachypneic, and febrile (38.5°C). On auscultation, inspiratory crackles were heard in the bilateral lung bases. Her bilateral lower legs showed pitting edema with many scratch scars, and the left leg was erythematous and hot. Pharyngeal redness, white moss, lymphadenopathy, arthritis, sensory disorders, and purpura were absent. Initial laboratory test results revealed an elevated white blood cell count (14,600/μl), an elevated C‐reactive protein level (11.6 mg/dl), and slightly decreased haemoglobin level (10.6 g/dl). The serum blood urea nitrogen, creatinine, and brain natriuretic peptide levels were 22 mg/dl, 1.18 mg/dl, and 269 pg/ml, respectively. The activated partial thromboplastin time (25.8 s) and prothrombin time‐international normalized ratio (0.95) were within normal ranges. Further investigation revealed a high anti‐streptolysin‐O (ASLO) titre (950 IU/ml) and low complement component‐3 level (62 mg/dl). The complement component‐4 level was within the normal range, and test results for immunoglobulin (Ig) G, IgA, IgM, antinuclear antibody, perinuclear ANCA (p‐ANCA), cytoplasmic ANCA(c‐ANCA), and anti‐glomerular basement membrane antibody were all negative. Urinalysis revealed 3+ protein, 2+ hematuria, and granular casts. Chest radiography showed an enlarged heart shadow and bilateral diffuse infiltrates with geographic distribution (Figure 1A). Computed tomography revealed multifocal diffuse consolidation and ground‐glass opacities, predominantly in the bilateral upper lung lobes (Figure 1B). Echocardiography showed a preserved left ventricular ejection fraction and no valvular disease or vegetation.

Laboratory data, including high titers of ASLO, worsening renal function, and complement component‐3 hypocomplementemia, prompted us to diagnose post‐streptococcal acute glomerulonephritis (PSAGN) caused by cellulitis of the left lower leg. The patient was treated with intravenous cefazolin (3 g/day) and furosemide; however, her respiratory symptoms deteriorated. To investigate the possibility of diffuse alveolar haemorrhage and rule out other pulmonary infections, bronchoscopy was performed under intubation. Bronchoalveolar lavage fluid from the right superior lobar bronchus was red and progressively darkened from the first to the third time owing to bleeding, which was consistent with diffuse alveolar haemorrhage (Figure 1C). Bronchoalveolar lavage fluid cytology did not reveal any malignant cells but revealed hemosiderin‐laden macrophages, and no microorganisms were grown on culture.

These findings supported the diagnosis of PRS secondary to a skin infection. The patient was treated with intravenous methylprednisolone pulse therapy (1 g/day) for three consecutive days, which resulted in significant improvement in the respiratory symptoms and chest X‐ray findings (Figure 1D). Moreover, the serum creatinine level decreased to the normal range. On day 4 of therapy, the patient was switched to oral steroids, which were tapered every 3 days for a total of 15 days (40, 20, 10, and 5 mg). The patient was discharged 15 days after admission. About 1 month follow‐up after steroid pulse therapy, she did not complain of respiratory symptoms. The serum ASLO level decreased to 410 IU/ml, and the haemoglobin level increased to 12.3 g/dl. In addition, the serum creatinine level, C‐reactive protein level, and chest X‐ray findings were normalized.

## DISCUSSION

This case highlights two important clinical lessons: (1) the patient developed PRS due to a streptococcal infection and (2) the patient was successfully treated with short‐term steroid therapy and remitted without recurrence.

Only a handful of cases of PRS caused by streptococcal infections have been reported.^2^, ^3^, ^4^ Most patients developed PRS after streptococcal pharyngitis,^2^, ^3^ and one developed PRS secondary to a skin infection.^4^ Generally, PSAGN manifests approximately 2 weeks after a skin infection, and the same period is required for the onset of PRS.^4^, ^5^ Our patient has always had many scratch scars on her bilateral lower legs due to atopic dermatitis, and the exact date of onset of the skin infection was unclear. The mechanism underlying PRS caused by streptococcal infections warrants further investigation. However, an immunological mechanism has been proposed. Antigens such as nephritis‐associated plasmin receptor and streptococcal exotoxin are involved in the pathogenesis of PSAGN; these antigens have the potential to form immune complexes and injure the alveolar basement membrane.^3^ Our patient had severe respiratory symptoms on presentation. Additionally, the patient was obese and could not maintain a prone position; therefore, renal and lung biopsies could not be performed. However, the clinical course, including a significant response to steroids, supports the involvement of immunological mechanisms.

Short‐term high‐dose steroid monotherapy may be sufficient to treat PRS caused by streptococcal infection. Steroids and immunosuppressive drugs (e.g., cyclophosphamide and azathioprine) and plasma exchange, are potential treatment options for PRS. However, PRS generally has poor prognosis and requires long‐term treatment.^1^ Although the treatment strategy for PRS is not well established, most cases of PRS caused by streptococcal infection remain in remission with steroid monotherapy.^2^ Since PSAGN often resolves within 4–14 days,^5^ we completed oral steroid therapy in approximately 2 weeks. Thus, the systemic steroid administration period was shorter than that in patients with other autoimmune diseases.

In conclusion, streptococcal infection is a relatively rare cause of PRS, and short‐term high‐dose steroid monotherapy may be sufficient for its treatment.

## AUTHOR CONTRIBUTIONS

Junki Shimokawa drafted the original manuscript. Hiroaki Nagano and Yoshihiko Raita supervised the conduct of this report. All authors reviewed the manuscript draft and revised it critically for intellectual content. All authors approved the final version of the manuscript to be published.

## CONFLICT OF INTEREST

None declared.

## ETHICS STATEMENT

The authors declare that appropriate written informed consent was obtained for the publication of this manuscript and accompanying images.

## Data Availability

The data that support the findings of this study are available from the corresponding author upon reasonable request.
